# Faithful Explanation Regeneration via Cauchy–Schwarz Mixture Information Bottleneck

**DOI:** 10.3390/e28080901

**Published:** 2026-08-10

**Authors:** Ziyang Wang, Junliang Du

**Affiliations:** 1 Department of Electronic Information Engineering, Glasgow College Hainan, University of Electronic Science and Technology of China, Hainan Lingshui Li’an International Education Innovation Pilot Zone, Lingshui 572400, China; 2023300904008@std.uestc.edu.cn; 2 AI Institute, Shanghai Jiao Tong University, Shanghai 200240, China

**Keywords:** large language models, free-text explanations, faithful explanation regeneration, information bottleneck, Cauchy–Schwarz divergence, Gaussian mixture prior

## Abstract

Large pretrained language models can generate fluent free-text explanations for natural language reasoning tasks, but these explanations may contain redundant, irrelevant, or unsupported information. In this paper, we propose a faithful explanation regeneration framework based on a Cauchy–Schwarz mixture information bottleneck. The proposed method compresses noisy explanations into a structured bottleneck representation while preserving task-relevant and decision-supporting information. Instead of using a unimodal Gaussian prior, we introduce a Gaussian mixture prior and employ the Cauchy–Schwarz divergence as a tractable compression regularizer. Furthermore, a faithfulness-aware objective is introduced to encourage the learned representation to remain aligned with the task decision. Experiments on free-text explanation benchmarks demonstrate that the proposed method improves explanation quality, conciseness, and faithfulness while providing an information-theoretic compression–preservation framework.

## 1. Introduction

Deep learning models have achieved remarkable success in a wide range of applications, including medical image analysis and distributed intelligent systems [1,2]. However, despite their strong predictive performance, modern deep learning models still face challenges related to reliability, interpretability, and trustworthy decision-making. In recent years, large language models (LLMs) have shown remarkable capabilities in generating natural language explanations for question answering, natural language inference, commonsense reasoning, and other language understanding tasks [3,4,5,6]. Free-text explanations are more flexible and human-readable than extractive rationales because they can describe implicit commonsense knowledge, causal relations, and intermediate reasoning steps in natural language [7,8]. However, the fluency of an explanation does not necessarily imply its reliability. LLM-generated explanations may contain redundant statements, irrelevant details, factual inconsistencies, or hallucinated reasoning that is not truly supportive of the task decision [9,10,11]. Therefore, explanation generation should aim not only to produce fluent text but also to regenerate explanations that are concise, sufficient, and faithful to the underlying decision.

A desirable free-text explanation should satisfy several complementary requirements. First, it should be concise, removing redundant or off-topic content that may obscure the main rationale. Second, it should be sufficient, preserving the information needed to justify the relationship between the input context and the output decision. Third, it should be faithful, in the sense that the explanation should support the task decision rather than merely provide a plausible post hoc narrative [8,10]. These requirements naturally suggest an information-theoretic view: explanation regeneration can be formulated as a problem of extracting a compact representation from a noisy explanation while preserving decision-relevant information. In this sense, the information bottleneck principle provides a principled framework for balancing compression and preservation [12,13].

Recent studies have explored information-theoretic objectives for natural language processing, including representation learning, rationale extraction, summarization, and explanation generation [14,15,16,17]. In particular, the information bottleneck principle has been used to refine machine-generated explanations by compressing initial explanation hypotheses while preserving predictive information about the explained task sample [17]. These works demonstrate the promise of bottleneck-based explanation regeneration. Nevertheless, two important issues remain underexplored. First, existing bottleneck formulations often regularize latent explanation representations toward a unimodal Gaussian prior. Such a prior may be too restrictive for explanation representations, since free-text explanations can contain diverse semantic components, including task-relevant rationales, redundant descriptions, contradictory statements, and hallucinated content. Second, most existing objectives mainly emphasize conciseness and sufficiency, while the faithfulness of regenerated explanations to the task decision is not explicitly modeled.

To address these issues, we propose a faithful explanation regeneration framework based on a Cauchy–Schwarz mixture information bottleneck. The key idea is to compress noisy machine-generated explanations into a structured bottleneck representation governed by a mixture-of-Gaussian prior and to use Cauchy–Schwarz divergence [18,19] as a tractable information-theoretic regularizer for matching the aggregated posterior of bottleneck representations to the mixture prior. Unlike a unimodal Gaussian bottleneck, the proposed mixture bottleneck is able to model the multimodal structure of explanation representations. Moreover, the Cauchy–Schwarz divergence admits a natural closed-form computation for Gaussian mixtures and has been widely used as an information-theoretic divergence measure [18,20,21]. This makes it particularly suitable for regularizing mixture-distributed bottleneck representations.

In addition to the mixture bottleneck, we introduce a faithfulness-aware regeneration objective. Instead of only requiring the bottleneck representation to support language generation, we encourage it to preserve decision-supporting information for the task output. Specifically, a lightweight verifier is used to predict the task decision from the input context and the learned bottleneck representation. The role of the verifier is to encourage the bottleneck representation to retain task-relevant decision-supporting information. It should not be interpreted as a direct causal faithfulness evaluator of the generated explanation, since the verifier is trained using task labels and therefore measures task alignment rather than complete explanation-level faithfulness. This objective explicitly encourages the regenerated explanation to remain aligned with the task decision, complementing conventional objectives for conciseness and sufficiency. The resulting framework balances three goals: compressing noisy explanation hypotheses, preserving predictive information about the task sample, and promoting faithfulness to the task decision.

The main contributions of this paper are summarized as follows:

- We formulate faithful explanation regeneration as a Cauchy–Schwarz mixture information bottleneck problem. The proposed bottleneck introduces a mixture-of-Gaussian prior to model the multimodal structure of explanation representations and uses Cauchy–Schwarz divergence as a tractable information-theoretic compression regularizer.
- We introduce a faithfulness-aware regeneration objective that encourages the learned bottleneck representation to support the task decision, thereby promoting explanations that are not only concise and sufficient but also decision-faithful.
- We conduct experiments on free-text explanation benchmarks to evaluate the proposed framework. The results show that our method improves explanation quality under automatic metrics, verifier-based faithfulness evaluation, and human-oriented criteria. Ablation studies further demonstrate the effectiveness of the proposed mixture bottleneck and faithfulness-aware objective.

## 2. Related Work

### 2.1. Free-Text Explanation Generation

Free-text explanations have been widely studied as a natural and human-readable form of model explanation. Unlike extractive rationales, which select input tokens or spans as evidence, free-text explanations can describe implicit commonsense knowledge, causal relations, and intermediate reasoning steps that are not explicitly contained in the input. Early work introduced explanation datasets for different NLP tasks, including science question answering, fact verification, commonsense validation, and defeasible inference [22,23,24,25,26]. These datasets support supervised learning of models that generate explanations together with task outputs.

Recent progress in large pretrained language models has shifted the field toward prompting-based explanation generation. Instead of training task-specific explanation models, prompting methods elicit explanations directly from large language models under zero-shot or few-shot settings. This paradigm is attractive because it reduces the need for task-specific annotation and can produce fluent reasoning-like text. However, prompting-based explanations may still contain irrelevant details, unsupported statements, or logically inconsistent reasoning. To address these limitations, recent studies have explored explanation filtering, self-correction, recursive prompting, and regeneration strategies [17,27,28]. Our work is related to explanation regeneration but focuses on learning a structured information bottleneck representation that supports concise and faithful regeneration.

### 2.2. Faithfulness and Evaluation of Explanations

A major challenge in free-text explanation generation is that a plausible explanation is not necessarily a faithful one. Faithfulness requires that the explanation genuinely supports the model decision or task output, rather than merely providing a convincing post hoc justification [10]. This issue is particularly important for large language models, because they may generate fluent explanations even when the explanation is inconsistent with the input, the answer, or the actual reasoning process [29]. Therefore, explanation quality should be assessed from multiple perspectives, including fluency, factuality, sufficiency, conciseness, and decision support.

Existing evaluation protocols combine automatic metrics and human judgments. Reference-based metrics, including BLEU [30], ROUGE [31], CIDEr [32], and BERTScore [33], are commonly used to measure lexical or semantic similarity to human-written explanations. However, these metrics are imperfect for open-ended explanations because multiple valid explanations may differ substantially in wording and reasoning structure. Human evaluation can better capture explanation-level properties, but it is expensive and may suffer from subjectivity. Recent work therefore emphasizes the need for more reliable faithfulness-oriented evaluation and training signals [8,9,10]. In this paper, we introduce a verifier-based faithfulness objective that encourages the bottleneck representation to support the task decision during training.

### 2.3. Information Bottleneck and Latent-Variable Modeling for Text Generation

The information bottleneck principle provides a principled framework for learning compressed representations that preserve task-relevant information [12]. It has inspired a variety of objectives in representation learning, summarization, rationale extraction, and explanation generation [34]. In NLP, bottleneck-based methods have been used to control conciseness in rationale extraction, sentence summarization, scientific document summarization, and controllable generation [14,15,16,35]. Explanation Regeneration via Information Bottleneck (EIB) formulates explanation refinement as an information bottleneck problem and demonstrates that compressing noisy machine-generated explanations while preserving task-relevant information can improve explanation conciseness and sufficiency [17]. However, EIB relies on a conventional bottleneck formulation with a unimodal Gaussian prior and mainly focuses on compression and sufficiency. In contrast, our work introduces a mixture-based bottleneck with a Cauchy–Schwarz divergence regularizer to model heterogeneous explanation representations, and it further incorporates a faithfulness-aware objective to encourage decision-supporting representations.

A related line of work studies usable information under computational constraints, which replaces exact mutual information estimation with model-dependent predictive information [36,37]. This view is suitable for text generation, where direct mutual information estimation is difficult due to high-dimensional representations and variable-length sequences. In our framework, we adopt a predictive preservation surrogate to ensure that the bottleneck representation remains informative about the task sample.

Beyond text generation, latent-variable modeling has shown that the choice of prior distribution strongly affects the structure and expressiveness of learned representations. Standard variational autoencoders often use a unimodal Gaussian prior [38,39], while more expressive priors, such as mixture priors or aggregated-posterior matching, can better capture multimodal data structure [40,41,42]. Motivated by this idea, we introduce a mixture-of-Gaussian prior for explanation bottlenecks. Instead of forcing all explanation representations toward a single Gaussian prior, the proposed bottleneck allows multiple latent modes corresponding to different semantic evidence and noise patterns in machine-generated explanations.

## 3. Method

### 3.1. Problem Formulation

Let *z* denote a task sample to be explained. In this work, we consider common free-text explanation settings where *z* consists of an input context *c* and a task output *y*, i.e.,

(1) z=(c,y).

For example, in commonsense question answering, *c* may contain a question and candidate answers, while *y* denotes the selected answer [43]. In natural language inference, *c* may contain a premise and a hypothesis, while *y* denotes the inference label. Given *z*, a large pretrained language model can generate an initial free-text explanation *x* through prompting:

(2) x=PLM(S(c,y)),

where S(·) is a task-dependent prompting function. Although such an explanation is often fluent and informative, it may contain redundant, irrelevant, or hallucinated content. The goal of explanation regeneration is therefore to transform the initial explanation *x* into a refined explanation $x^{\prime }$ that is concise, sufficient, and faithful to the task decision.

We formulate explanation regeneration as an information bottleneck problem. Specifically, the refiner first maps the initial explanation *x* into a continuous bottleneck representation *T* through a stochastic encoder:

(3) $$q_{\theta }\left(T|x\right),$$

where θ denotes the parameters of the refiner. The bottleneck representation *T* is expected to satisfy two requirements. First, it should be compact with respect to the initial explanation *x*, so that redundant or noisy information is suppressed. Second, it should preserve information that is useful for explaining the task sample *z* and supporting the output *y*. Based on *T*, a generator then produces the regenerated explanation:

(4) $$x^{\prime }∼p_{\delta }\left(x^{\prime }|T,x,z\right),$$

where δ denotes the parameters of the generator.

From an information-theoretic perspective, the desired bottleneck representation should balance compression and preservation. Following the information bottleneck principle, a generic explanation bottleneck objective can be written as

(5) $$L_{\mathrm{IB}}=\beta I\left(X;T\right)-I\left(T;Z\right),$$

where I(X;T) measures the amount of information retained from the initial explanation and I(T;Z) measures the predictive information preserved about the task sample. The coefficient β>0 controls the strength of compression.

In practice, directly optimizing these mutual information terms is difficult for free-text explanation regeneration. Conventional variational bottleneck formulations often replace the compression term with a KL divergence between the samplewise posterior and a standard Gaussian prior [13]. In contrast, we introduce a Cauchy–Schwarz mixture bottleneck, which regularizes the aggregated posterior toward a Gaussian mixture prior and provides a more flexible information-theoretic compression surrogate for explanation representations.

The full explanation regeneration framework combines bottleneck learning and sequence generation. Given a training tuple $\left(z,x,x^{*}\right)$, where $x^{*}$ denotes a reference explanation, the generator is optimized to maximize the conditional likelihood of the reference explanation:

(6) $$L_{\mathrm{gen}}=-E_{T∼q_{\theta }\left(T|x\right)}\left[\log p_{\delta }\left(x^{*}|T,x,z\right)\right].$$

Here, *x* is the noisy or imperfect explanation hypothesis, while $x^{*}$ is the target explanation. During inference, the model takes a task sample *z* and an initial explanation *x* as input, infers the bottleneck representation *T*, and autoregressively generates a refined explanation $x^{\prime }$.

**Figure 1:**
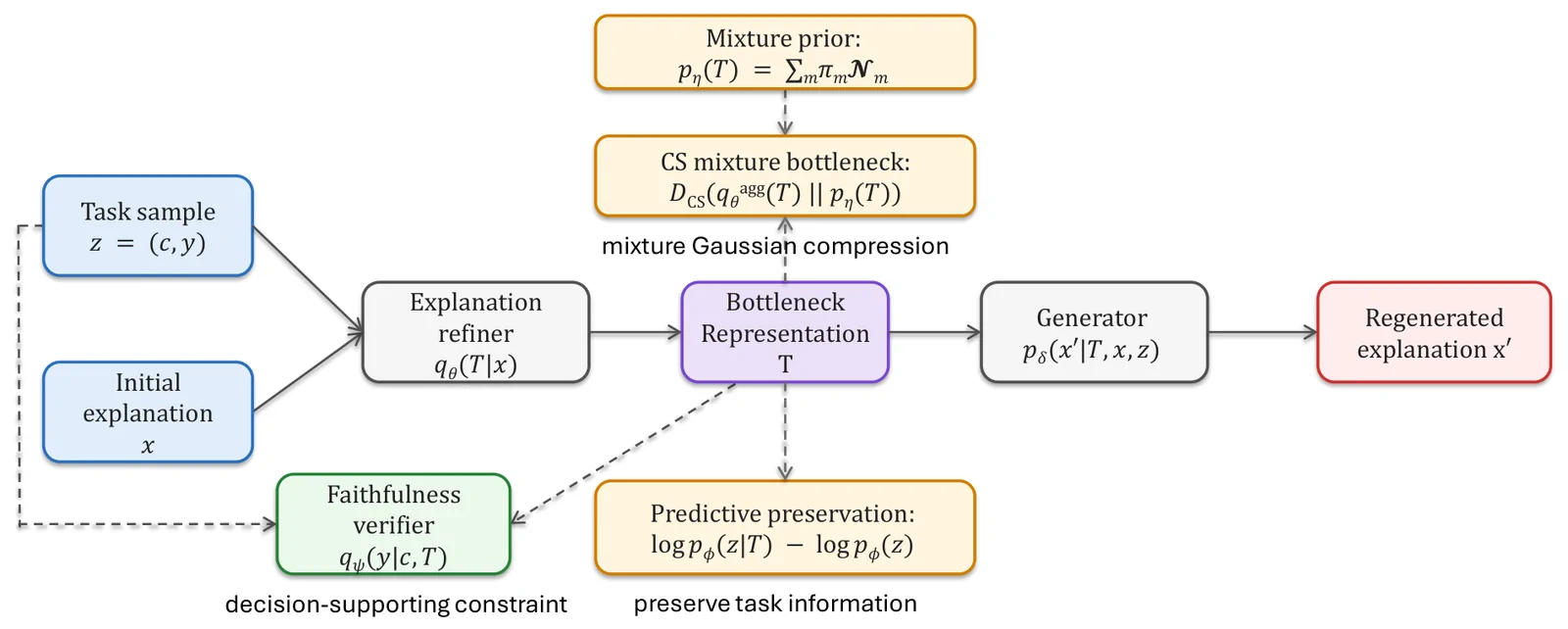
Overall framework of the proposed faithful explanation regeneration method. Given a task sample z=(c,y) and an initial explanation *x*, the refiner encodes *x* into a bottleneck representation *T*. The CS mixture bottleneck regularizes the aggregated posterior toward a mixture prior, while the predictive preservation objective encourages *T* to retain task-relevant information. A faithfulness verifier further encourages *T* to support the task decision, and the generator produces the regenerated explanation $x^{\prime }$ conditioned on *T*, *x*, and *z*.

Overall, our objective is to learn a bottleneck representation that removes unnecessary information from the initial explanation, preserves predictive information about the task sample, and supports faithful regeneration of the final explanation. The overall framework is illustrated in Figure 1. The next subsection describes the predictive information preservation term, while the following subsections introduce the proposed Cauchy–Schwarz mixture bottleneck and the faithfulness-aware objective.

### 3.2. Predictive Information Preservation

The bottleneck representation *T* should not only compress the initial explanation *x* but also retain information useful for explaining the task sample *z*. Directly estimating the mutual information I(T;Z) is challenging in free-text explanation generation because both *T* and *z* may involve high-dimensional continuous representations and variable-length text sequences. Therefore, we adopt a model-based predictive information preservation surrogate.

Let $p_{\phi }$ be a computationally bounded autoregressive language model used to measure how much predictive information *T* provides about the task sample *z*. The preservation term is defined as the improvement in predictive log-likelihood when the bottleneck representation is available:

(7) $${\hat{I}}_{\phi }\left(T;Z\right)=E_{z,T}\left[\log p_{\phi }\left(z|T\right)-\log p_{\phi }\left(z\right)\right].$$

Equivalently, this term can be viewed as a model-dependent reduction in coding cost:

(8) $${\hat{I}}_{\phi }\left(T;Z\right)=H_{\phi }\left(Z\right)-H_{\phi }\left(Z|T\right),$$

where

(9) $$H_{\phi }\left(Z\right)=E_{z}\left[-\log p_{\phi }\left(z\right)\right],$$

and

(10) $$H_{\phi }\left(Z|T\right)=E_{z,T}\left[-\log p_{\phi }\left(z|T\right)\right].$$

If *T* contains useful information about the task sample, then conditioning on *T* should reduce the predictive uncertainty of *z*, leading to a larger value of ${\hat{I}}_{\phi }\left(T;Z\right)$.

In practice, the bottleneck representation *T* is used as a continuous prefix or conditioning signal for the predictive model $p_{\phi }$. The task sample *z* is linearized into a text sequence according to the task format. For example, in question answering, *z* may be represented by the concatenation of the question and the answer; in natural language inference, *z* may be represented by the premise, hypothesis, and label. The predictive preservation loss is then written as

(11) $$L_{\mathrm{pres}}=-E_{z,T}\left[\log p_{\phi }\left(z|T\right)-\log p_{\phi }\left(z\right)\right].$$

Since $\log p_{\phi }\left(z\right)$ does not depend on the bottleneck encoder parameters once $p_{\phi }$ is fixed, optimizing this objective mainly encourages *T* to increase the conditional likelihood $\log p_{\phi }\left(z|T\right)$. This prevents the bottleneck representation from discarding information that is necessary for explaining the task sample.

### 3.3. Cauchy–Schwarz Mixture Information Bottleneck

Given an initial machine-generated explanation *x*, the refiner encodes it into a bottleneck representation *T*. Following the information bottleneck principle, this representation should discard redundant and noisy information in *x* while retaining information useful for explanation regeneration. A common choice is to regularize the posterior distribution of the bottleneck variable toward a standard Gaussian prior. However, such a unimodal prior can be overly restrictive for free-text explanations. In practice, explanation representations may exhibit heterogeneous semantic patterns, corresponding to task-relevant rationales, redundant descriptions, unsupported claims, and potentially contradictory statements. This motivates a more expressive latent prior for explanation bottlenecks.

Let the encoder parameterize a samplewise posterior distribution

(12) $$q_{\theta }\left(t|x\right)=N\left(t;\mu_{\theta }\left(x\right),\mathrm{diag}\left(\sigma_{\theta }^{2}\left(x\right)\right)\right),$$

where $\mu_{\theta }\left(x\right)$ and $\sigma_{\theta }^{2}\left(x\right)$ are produced by the refiner network. Instead of imposing a standard Gaussian prior, we introduce a mixture-of-Gaussian prior:

(13) $$p_{\eta }\left(t\right)=\sum_{m=1}^{M}\pi_{m}N\left(t;\nu_{m},\Lambda_{m}\right),$$

where $\pi_{m}\geq 0$, $\sum_{m=1}^{M}\pi_{m}=1$, and $\nu_{m}$ and $\Lambda_{m}$ denote the mean and covariance of the *m*th mixture component. This prior provides a more flexible distributional structure for modeling multiple types of explanation semantics and noise patterns.

Rather than match each individual posterior $q_{\theta }\left(t|x\right)$ to the prior, we regularize the aggregated posterior over a mini-batch. Given a mini-batch ${\left\{x_{i}\right\}}_{i=1}^{B}$, the aggregated posterior is approximated by

(14) $$q_{\theta }^{\mathrm{agg}}\left(t\right)=\frac{1}{B}\sum_{i=1}^{B}q_{\theta }\left(t|x_{i}\right).$$

Since each $q_{\theta }\left(t|x_{i}\right)$ is Gaussian, the aggregated posterior is also a Gaussian mixture. This makes it natural to compare $q_{\theta }^{\mathrm{agg}}\left(t\right)$ with the mixture prior $p_{\eta }\left(t\right)$ at the distribution level.

To obtain a tractable information-theoretic compression regularizer, we employ the Cauchy–Schwarz divergence [18,19] between the aggregated posterior and the mixture prior:

(15) $$D_{\mathrm{CS}}\left(q_{\theta }^{\mathrm{agg}}∥p_{\eta }\right)=-\log\frac{\int q_{\theta }^{\mathrm{agg}}\left(t\right)p_{\eta }\left(t\right)dt}{\sqrt{\int {\left(q_{\theta }^{\mathrm{agg}}\left(t\right)\right)}^{2}dt\int p_{\eta }^{2}\left(t\right)dt}}.$$

Equivalently,

(16) $$D_{\mathrm{CS}}\left(q_{\theta }^{\mathrm{agg}}∥p_{\eta }\right)=-\log\langle q_{\theta }^{\mathrm{agg}},p_{\eta }\rangle +\frac{1}{2}\log\langle q_{\theta }^{\mathrm{agg}},q_{\theta }^{\mathrm{agg}}\rangle +\frac{1}{2}\log\langle p_{\eta },p_{\eta }\rangle ,$$

where 〈f,g〉=∫f(t)g(t)dt denotes the density inner product.

The advantage of this regularizer is that all three overlap terms admit closed-form computation when both distributions are Gaussian mixtures. Specifically, let

(17) $$q\left(t\right)=\sum_{i=1}^{B}w_{i}N\left(t;\mu_{i},\Sigma_{i}\right),\;p\left(t\right)=\sum_{m=1}^{M}\pi_{m}N\left(t;\nu_{m},\Lambda_{m}\right),$$

where $w_{i}=1/B$. Then, the cross-density inner product is

(18) $$\langle q,p\rangle =\sum_{i=1}^{B}\sum_{m=1}^{M}w_{i}\pi_{m}N\left(\mu_{i};\nu_{m},\Sigma_{i}+\Lambda_{m}\right).$$

Similarly,

(19) $$\langle q,q\rangle =\sum_{i=1}^{B}\sum_{j=1}^{B}w_{i}w_{j}N\left(\mu_{i};\mu_{j},\Sigma_{i}+\Sigma_{j}\right),$$

and

(20) $$\langle p,p\rangle =\sum_{m=1}^{M}\sum_{n=1}^{M}\pi_{m}\pi_{n}N\left(\nu_{m};\nu_{n},\Lambda_{m}+\Lambda_{n}\right).$$

Therefore, the CS divergence provides a closed-form and differentiable compression objective for the proposed mixture bottleneck.

The resulting CS mixture bottleneck loss is defined as

(21) $$L_{\mathrm{CS}\text{-}\mathrm{MIB}}=D_{\mathrm{CS}}\left(q_{\theta }^{\mathrm{agg}}\left(t\right)∥p_{\eta }\left(t\right)\right).$$

Compared with a unimodal Gaussian bottleneck, this objective encourages the explanation representation space to remain compact while preserving a flexible multimodal structure. Such a design is particularly suitable for explanation regeneration, where different samples may require different forms of evidence selection, redundancy removal, and hallucination suppression.

### 3.4. Faithfulness-Aware Explanation Regeneration

The predictive preservation term encourages the bottleneck representation *T* to retain information about the task sample *z*. However, preserving information about the task sample does not necessarily guarantee that the regenerated explanation is faithful to the task decision. A regenerated explanation may be concise and fluent but still fail to provide evidence that truly supports the output *y*. To address this issue, we introduce a faithfulness-aware objective that explicitly encourages the bottleneck representation to preserve decision-supporting information.

Let z=(c,y), where *c* denotes the input context and *y* denotes the task output. We introduce a lightweight verifier $q_{\psi }$ that predicts the task output from the context *c* and the bottleneck representation *T*:

(22) $$q_{\psi }\left(y|c,T\right),$$

where ψ denotes the verifier parameters. The verifier is trained jointly with the explanation regeneration framework. Since the task output *y* is already available in explanation datasets, this objective does not require additional human annotation. The faithfulness-aware loss is defined as

(23) $$L_{\mathrm{faith}}=-E_{T∼q_{\theta }\left(T|x\right)}\left[\log q_{\psi }\left(y|c,T\right)\right].$$

Minimizing this loss encourages the bottleneck representation to retain information that is predictive of the task decision. In other words, the learned representation is not only optimized for regenerating a fluent explanation but also constrained to preserve decision-supporting information associated with the output being explained. However, this latent-level alignment should not be interpreted as a complete causal faithfulness guarantee, since the verifier is trained using task annotations and may still capture predictive patterns that are not necessarily equivalent to human-interpretable reasoning.

This design provides a practical way to incorporate faithfulness into explanation regeneration. Directly imposing a faithfulness loss on the generated explanation $x^{\prime }$ is difficult because $x^{\prime }$ is a discrete sequence and the corresponding objective may require reinforcement learning or non-differentiable re-ranking. By contrast, the proposed verifier operates on the continuous bottleneck representation *T*, making the objective differentiable and easy to optimize by back-propagation. Moreover, this latent-level faithfulness constraint is complementary to the generation loss: the generation loss teaches the model to produce human-like explanations, while the faithfulness loss encourages the internal explanatory representation to remain aligned with the task decision.

For implementation, the verifier can be built as a small prediction head on top of the concatenation of the context representation and the bottleneck representation. Let $r_{c}$ be the contextual representation of *c*, obtained from the same frozen or trainable encoder used in the refiner. The verifier can be parameterized as

(24) $$q_{\psi }\left(y|c,T\right)=\mathrm{softmax}\left(W_{\psi }\left[r_{c};\mathrm{Pool}\left(T\right)\right]+b_{\psi }\right),$$

where [·;·] denotes concatenation, Pool(·) is a pooling operation over the bottleneck vectors, and $W_{\psi }$ and $b_{\psi }$ are learnable parameters. For classification tasks such as natural language inference, *y* is a class label. For question-answering tasks, *y* can be represented as the selected answer option or a normalized answer label.

The proposed faithfulness-aware objective is not intended to replace human evaluation of explanation quality. Instead, it provides an explicit training signal that encourages the learned bottleneck to be decision-supporting. As a result, the regenerated explanation is expected to better satisfy the faithfulness requirement in addition to conciseness and sufficiency.

### 3.5. Overall Objective

The proposed framework consists of four complementary components: explanation generation, predictive information preservation, Cauchy–Schwarz mixture bottleneck compression, and faithfulness-aware decision support. Given a training tuple $\left(z,x,x^{*}\right)$, where z=(c,y) is the task sample, *x* is the initial noisy explanation, and $x^{*}$ is the reference explanation, the stochastic refiner first produces a bottleneck representation

(25) $$T∼q_{\theta }\left(T|x\right).$$

The generator then regenerates the explanation by modeling

(26) $$p_{\delta }\left(x^{*}|T,x,z\right).$$

The generation loss is given by the negative conditional log-likelihood:

(27) $$L_{\mathrm{gen}}=-E_{T∼q_{\theta }\left(T|x\right)}\left[\log p_{\delta }\left(x^{*}|T,x,z\right)\right].$$

This term encourages the model to produce explanations that are close to human-written or high-quality target explanations.

The predictive information preservation loss is defined as

(28) $$L_{\mathrm{pres}}=-E_{z,T}\left[\log p_{\phi }\left(z|T\right)-\log p_{\phi }\left(z\right)\right].$$

This term encourages *T* to retain information that makes the task sample easier to predict under the computationally bounded predictive model $p_{\phi }$.

The proposed Cauchy–Schwarz mixture bottleneck loss is

(29) $$L_{\mathrm{CS}\text{-}\mathrm{MIB}}=D_{\mathrm{CS}}\left(q_{\theta }^{\mathrm{agg}}\left(T\right)∥p_{\eta }\left(T\right)\right),$$

where $q_{\theta }^{\mathrm{agg}}\left(T\right)$ is the mini-batch aggregated posterior and $p_{\eta }\left(T\right)$ is the mixture-of-Gaussian prior. This term regularizes the explanation bottleneck space by encouraging compactness while allowing multimodal latent structure.

The faithfulness-aware loss is

(30) $$L_{\mathrm{faith}}=-E_{T∼q_{\theta }\left(T|x\right)}\left[\log q_{\psi }\left(y|c,T\right)\right],$$

which encourages the bottleneck representation to support the task decision.

Combining these terms, the overall training objective is

(31) $$L_{\mathrm{total}}=L_{\mathrm{gen}}+\beta L_{\mathrm{CS}\text{-}\mathrm{MIB}}+\lambda L_{\mathrm{pres}}+\gamma L_{\mathrm{faith}},$$

where β, λ, and γ are non-negative hyperparameters controlling the strengths of bottleneck compression, predictive preservation, and faithfulness-aware supervision, respectively.

The four terms play distinct roles. The generation loss ensures that the regenerated explanation is fluent and aligned with the reference explanation. The CS mixture bottleneck loss removes redundant and noisy information from the initial explanation while maintaining a structured latent space. The predictive preservation loss prevents the bottleneck from discarding information needed to explain the task sample. The faithfulness-aware loss further encourages the learned representation to support the output decision. Together, these objectives enable the model to regenerate explanations that are concise, sufficient, and faithful.

During inference, given a task sample z=(c,y) and an initial explanation *x* generated by a pretrained language model, the refiner infers the bottleneck representation *T* from $q_{\theta }\left(T|x\right)$. The generator then autoregressively decodes the refined explanation:

(32) $$x^{\prime }=\arg\max_{\tilde{x}}p_{\delta }\left(\tilde{x}|T,x,z\right).$$

No reference explanation is required at inference time. The verifier can optionally be used to score or re-rank multiple generated candidates, but the main framework requires only the learned bottleneck representation and the generator to produce the final explanation.

## 4. Experiments

### 4.1. Experimental Setup

#### 4.1.1. Datasets

We evaluate the proposed framework on two representative free-text explanation benchmarks: ECQA and e-SNLI. ECQA is a commonsense question answering dataset where each instance contains a question, answer candidates, the correct answer, and a natural language explanation. e-SNLI extends natural language inference with human-written explanations, where each instance contains a premise, a hypothesis, an inference label, and a free-text explanation. These two datasets cover different explanation scenarios: commonsense explanation and entailment-based reasoning explanation.

Following the explanation regeneration setting, each task sample is denoted as z=(c,y), where *c* is the input context and *y* is the task output. For ECQA, *c* contains the question and answer candidates, while *y* is the selected answer. For e-SNLI, *c* contains the premise and hypothesis, while *y* is the inference label. The initial explanation *x* is generated by a pretrained language model using task-specific prompts, and the reference explanation $x^{*}$ is used as the target during training.

To train the regeneration model, we construct noisy explanation inputs from reference explanations using automatic perturbation operations, including repetition, replacement, negation alteration, sentence shuffling, and infilling. These perturbations provide a controlled evaluation protocol for isolating the effect of removing redundant, irrelevant, and inconsistent information from explanations. However, they do not fully capture the complete distribution of errors produced by real LLM-generated explanations, which may involve more complex factual hallucinations or reasoning failures.

#### 4.1.2. Baselines

We compare the proposed method with several representative baselines. The first baseline is Prompting, where a pretrained language model directly generates explanations from task-specific prompts without additional refinement [3,4]. We also consider Prompting–Filter, which generates multiple explanation candidates by prompting and selects the most plausible one using a filtering model. In addition, we include Supervised Generator, where a sequence-to-sequence or autoregressive language model is fine-tuned on target-domain explanation data. To compare with information bottleneck-based text compression methods, we include BottleSum, an information-bottleneck-based summarization method that compresses the input explanation into a shorter version [14]. We further compare our method with EIB, a closely related explanation regeneration method based on the information bottleneck principle, which compresses initial explanations and regenerates refined explanations using a bottleneck representation [17]. Finally, we evaluate two ablated variants of our method: Ours w/o Faith, which removes the faithfulness-aware objective, and Ours w/o CS-MIB, which replaces the proposed Cauchy–Schwarz mixture bottleneck with a standard Gaussian KL bottleneck. The full model, denoted as Ours, combines the Cauchy–Schwarz mixture information bottleneck with the faithfulness-aware regeneration objective.

#### 4.1.3. Evaluation Metrics

We evaluate generated explanations from three complementary perspectives. First, we assess reference-based generation quality using BLEU-4 [30], ROUGE-L [31], CIDEr [32], and BERTScore [33], which measure lexical or semantic similarity between generated explanations and reference explanations. Second, we evaluate diversity and conciseness using Distinct-1, Distinct-2, Novelty-1, Novelty-2, and average length. Distinct-*n* measures lexical diversity by computing the ratio of unique *n*-grams among all generated *n*-grams, where n=1 and n=2 correspond to unigram and bigram diversity, respectively. Novelty-*n* measures the proportion of generated *n*-grams that do not appear in the input context, indicating the amount of newly introduced information in the regenerated explanations. Average length provides a simple indicator of explanation conciseness.

Third, we evaluate faithfulness and decision support using verifier accuracy and verifier log-likelihood. The verifier predicts the task output *y* from the context and the generated explanation or bottleneck representation. A higher verifier score indicates stronger task alignment of the learned representation and provides an automatic proxy for faithfulness-related evaluation. For e-SNLI, we additionally report an NLI-based consistency score to estimate whether the generated explanation is consistent with the premise, hypothesis, and label.

#### 4.1.4. Implementation Details

The refiner and generator are initialized from GPT-2 Small. The bottleneck representation is implemented as a sequence of continuous prefix vectors. The predictive preservation model $p_{\phi }$ shares the same architecture as the generator but has a separate prediction head. The mixture prior contains M=8 Gaussian components unless otherwise specified. The covariance matrices are diagonal for computational efficiency. The model is trained with Adam using a learning rate of $5\times 10^{-5}$, batch size 32, and early stopping based on validation BERTScore and verifier score. The hyperparameters β, λ, and γ are selected from $\left\{10^{-5},10^{-4},10^{-3}\right\}$, {0.1,0.5,1.0}, and {0.1,0.5,1.0}, respectively.

### 4.2. Main Results

Table 1 reports the automatic evaluation results on ECQA. The proposed method achieves the best overall performance among unsupervised or weakly supervised refinement methods. Compared with direct prompting, our method substantially reduces the average explanation length while improving BERTScore, CIDEr, and verifier-based faithfulness. Compared with EIB, the proposed method achieves superior faithfulness scores and slightly improved reference-based metrics, suggesting that the CS mixture bottleneck and faithfulness-aware objective help preserve decision-supporting information while removing redundant content.

Table 2 reports the results on e-SNLI. The proposed method consistently improves over prompting-based baselines and the original bottleneck-based regeneration baseline. The improvement is particularly clear in verifier accuracy and NLI consistency, indicating that the generated explanations are more aligned with the task decision. The average length remains close to that of concise human explanations, suggesting that the proposed model does not improve faithfulness by simply generating longer explanations.

### 4.3. Evaluation on Naturally Generated LLM Explanations

To further evaluate the robustness of explanation regeneration under realistic conditions, we additionally conduct experiments using naturally generated LLM explanations. In contrast to the controlled perturbation setting, where noise is introduced manually, the initial explanations in this experiment are directly generated by a pretrained language model. The generated explanations are then provided as inputs to different regeneration methods, including EIB and the proposed approach. We evaluate the regenerated explanations using the same automatic metrics described above.

The results in Table 3 and Table 4 demonstrate that the proposed method remains effective when the initial explanations are directly generated by a pretrained language model rather than constructed through synthetic perturbations. Compared with EIB, our method achieves consistent improvements in both explanation generation quality and faithfulness-related evaluation metrics. These results indicate that the effectiveness of the proposed CS-MIB is not limited to controlled perturbation settings but can also generalize to explanations generated in a more realistic LLM generation scenario.

### 4.4. Ablation Study

To examine the contribution of each component, we conduct ablation studies on the proposed Cauchy–Schwarz mixture bottleneck and the faithfulness-aware objective. Table 5 summarizes the results. Removing the CS mixture bottleneck leads to lower BERTScore and Distinct scores, suggesting that the mixture bottleneck provides a more expressive and structured latent space for explanation compression. Removing the faithfulness objective mainly reduces verifier accuracy and NLI consistency, confirming that this objective contributes directly to decision-supporting explanations.

The preservation term is also important. Without predictive preservation, the model tends to produce shorter explanations but loses task-relevant information. This is reflected by lower BERTScore and verifier accuracy. Without bottleneck regularization, the model tends to copy or retain noisy content from the initial explanation, resulting in longer and less concise generations.

### 4.5. Effect of the Number of Mixture Components

We further analyze the effect of the number of Gaussian components in the mixture prior. Table 6 reports the results with M∈{1,4,8,16}. When M=1, the prior reduces to a unimodal Gaussian prior. Increasing the number of components improves both generation quality and verifier accuracy, indicating that explanation representations benefit from a multimodal latent structure. However, the improvement becomes saturated when *M* becomes too large, and M=8 provides a good balance between performance and computational cost.

### 4.6. Batch Size Sensitivity Analysis

We further analyze the sensitivity of CS-MIB to different batch sizes on ECQA. As shown in Table 7, the proposed method maintains stable performance across batch sizes ranging from 16 to 128. The default setting (B=32) achieves comparable performance to larger batch sizes, suggesting that CS-MIB does not rely on large mini-batches for effective optimization. As expected, the computational overhead increases with batch size due to the pairwise Gaussian interactions required by the closed-form Cauchy–Schwarz divergence. However, the additional cost remains moderate under the default setting.

### 4.7. Compression–Faithfulness Trade-Off

The proposed framework contains a compression coefficient β that controls the strength of the CS mixture bottleneck. To study the compression–faithfulness trade-off, we vary β and report average length, BERTScore, and verifier accuracy in Table 8. A very small β weakens compression and may retain redundant or noisy content from the initial explanation. A very large β overcompresses the bottleneck representation and may remove information necessary for supporting the task decision. The best performance is achieved with a moderate value of β, confirming the importance of balancing compression and preservation.

### 4.8. Human Evaluation

Since automatic metrics may not fully capture explanation quality, we conduct a human evaluation on a randomly sampled subset of ECQA and e-SNLI test examples. Following prior work, annotators evaluate explanations along five dimensions: grammaticality, factuality, new information, sufficiency, and conciseness. We additionally include a faithfulness criterion, asking whether the explanation provides evidence that supports the given task output.

Table 9 reports the human evaluation results. The proposed method obtains higher scores in sufficiency, conciseness, and faithfulness than prompting-based baselines. Compared with EIB, the proposed method shows the largest improvement in faithfulness, which is consistent with the verifier-based automatic evaluation.

### 4.9. Qualitative Visualization

**Figure 2:**
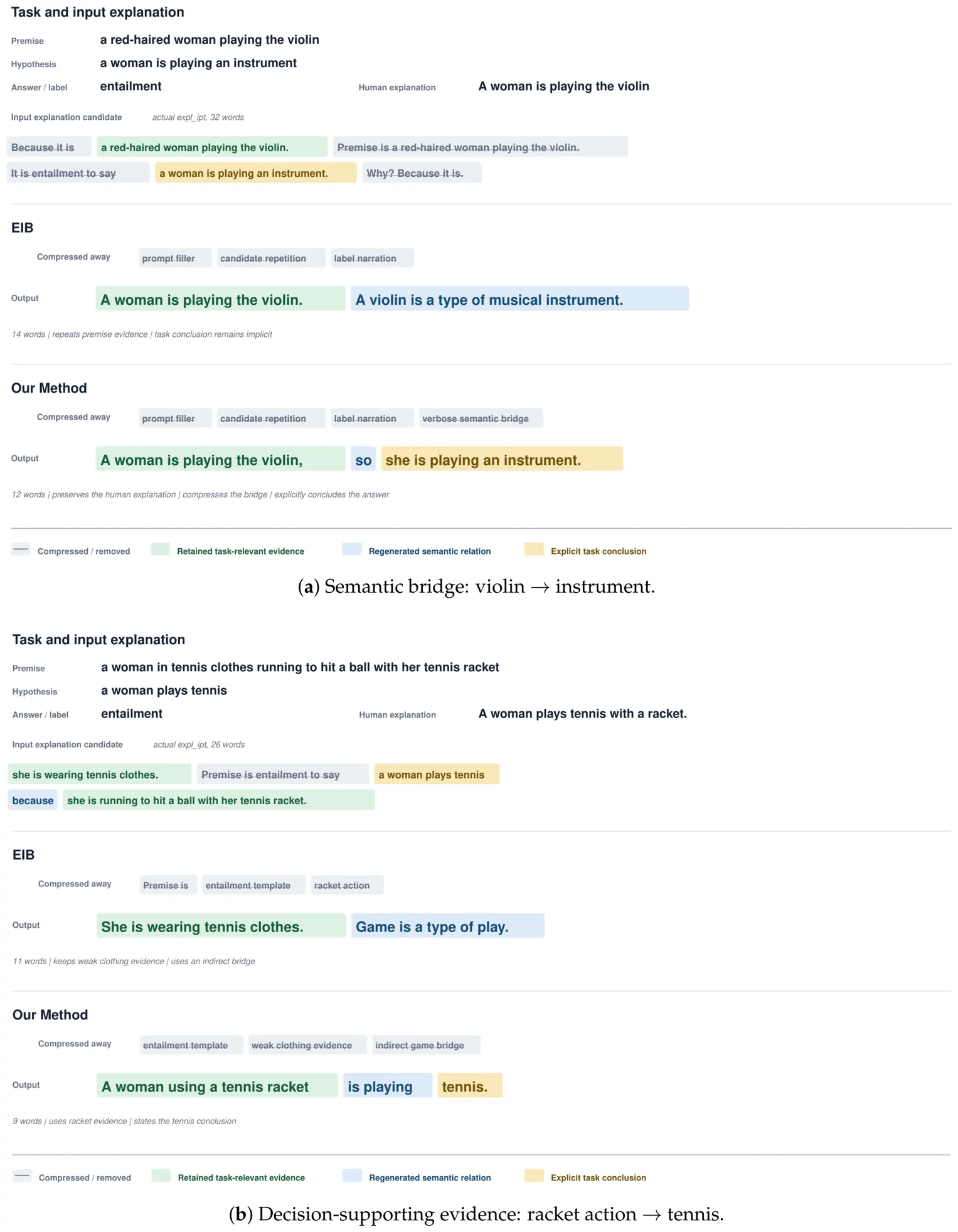
Qualitative visualization of explanation regeneration on semantic bridge and decision support. (**a**) The proposed method explicitly connects the retained evidence “playing the violin” with the task conclusion “playing an instrument”. (**b**) The proposed method focuses on the decision-supporting racket action, while EIB retains weaker clothing evidence and uses an indirect bridge.

**Figure 3:**
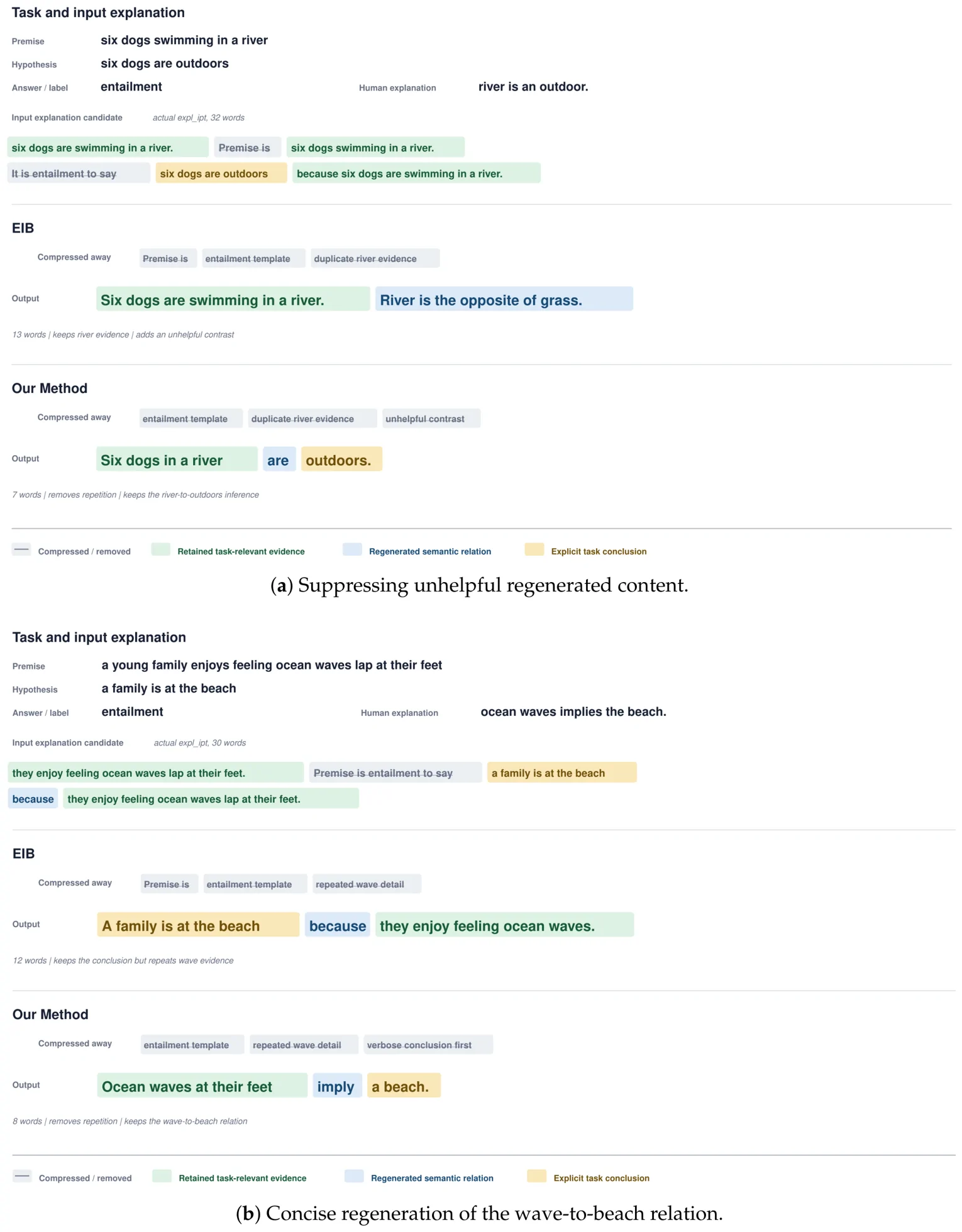
Qualitative visualization of explanation regeneration as it affects noise suppression and concise reasoning.(**a**) EIB keeps the river evidence but introduces an unhelpful contrast, whereas our method directly preserves the river-to-outdoors inference. (**b**) Our method removes repeated wave details and regenerates a concise semantic relation between ocean waves and the beach.

Figure 2 and Figure 3 provide qualitative comparisons between EIB and our method. The examples cover four representative error patterns: missing semantic bridges, weak decision-supporting evidence, unhelpful regenerated content, and redundant explanation patterns. Overall, EIB often shortens the input explanation and removes part of the prompt-style noise. However, it may still leave the task conclusion implicit, retain weaker evidence, or regenerate relations that are not directly useful for supporting the label. In contrast, our method more consistently preserves task-relevant evidence and regenerates explicit semantic relations that support the task decision.

These examples suggest that the improvement of our method is not merely due to shorter generations. Instead, the proposed CS mixture bottleneck and faithfulness-aware objective help the model suppress noisy explanation modes while retaining evidence that is directly useful for explaining the output label.

**Figure 4:**
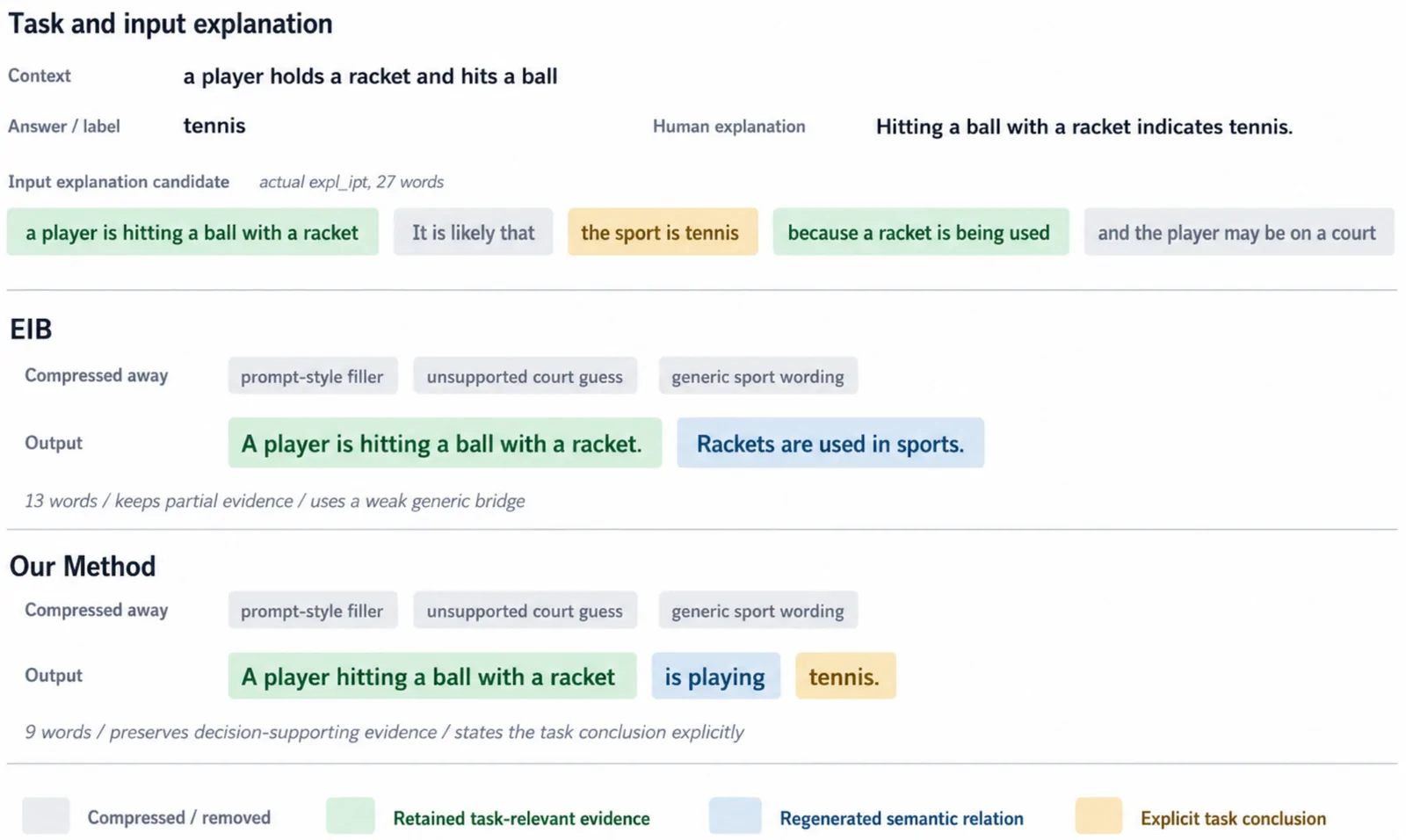
Qualitative comparison of explanation regeneration as it affects decision-supporting evidence preservation.The initial explanation is generated from the input context “a player holds a racket and hits a ball” with the task label “tennis”. EIB preserves part of the racket-related evidence but introduces a weak generic semantic bridge (“Rackets are used in sports”). In contrast, the proposed method retains the decision-supporting evidence (“hitting a ball with a racket”) and explicitly connects it to the task conclusion (“is playing tennis”). This example illustrates that the proposed method better preserves task-relevant information while reducing unsupported or less informative explanation patterns.

To further investigate whether the proposed faithfulness-aware objective preserves human-interpretable decision-supporting evidence, we provide an additional qualitative case study in Figure 4.

In this example, the task is to identify whether the described action corresponds to tennis. EIB preserves the core racket-related evidence but introduces a weak generic semantic bridge (“rackets are used in sports”), which does not directly connect the evidence to the task conclusion. In contrast, the proposed method retains the decision-supporting evidence (“hitting a ball with a racket”) and explicitly regenerates the semantic relation to the label (“is playing tennis”). This example illustrates that the proposed bottleneck representation can preserve task-relevant evidence while reducing unsupported or less informative reasoning patterns.

## 5. Conclusions and Future Work

In this paper, we proposed a faithful explanation regeneration framework based on a Cauchy–Schwarz mixture information bottleneck. The central idea is to compress noisy machine-generated explanations into a structured bottleneck representation while preserving task-relevant and decision-supporting information. In contrast to conventional bottleneck formulations that usually rely on a unimodal Gaussian prior, the proposed method introduces a Gaussian mixture prior to capture the multimodal structure of free-text explanation representations. To make this objective tractable, we employed the Cauchy–Schwarz divergence between the aggregated posterior and the mixture prior as a closed-form compression regularizer. In addition, we introduced a faithfulness-aware verifier objective to encourage the bottleneck representation to support the task decision. Experiments on ECQA and e-SNLI show that the proposed framework improves reference-based generation quality, conciseness, and verifier-based faithfulness compared with prompting-based and bottleneck-based baselines.

Despite these promising results, several limitations remain. First, the faithfulness constraint in our framework is primarily imposed at the latent representation level. Specifically, the verifier encourages the bottleneck representation to preserve decision-supporting information by predicting the task output from the context and the learned representation. However, such latent-level task alignment does not necessarily guarantee complete text-level faithfulness, as the decoder may introduce unsupported or imperfect information during the generation process. Moreover, since the verifier is trained using task annotations, it may still capture predictive shortcuts rather than the complete causal reasoning process underlying human-interpretable explanations. Therefore, the verifier-based objective should be interpreted as a faithfulness-related alignment signal rather than a complete causal faithfulness guarantee. Developing stronger intervention-based evaluation protocols and causal faithfulness objectives remains an important direction for future work.

## Figures and Tables

**Table 1:** Automatic evaluation on ECQA. Higher is better for all metrics except AvgLen (average length), where lower values indicate more concise explanations. The best performance is in bold.

Method	BERTScore	CIDEr	BLEU-4	Dist-1	Dist-2	Nov-2	AvgLen	Verifier Acc.
Supervised Generator	87.6	78.0	11.2	22.1	58.0	51.5	16.8	82.4
BottleSum	84.8	16.9	3.8	16.4	45.0	54.1	16.3	72.5
Prompting	84.4	14.5	3.2	11.5	34.5	54.7	27.5	70.8
Prompting–Filter	85.3	17.2	3.4	13.2	47.4	61.3	27.1	74.6
EIB	85.8	20.4	3.2	16.5	48.3	61.4	16.6	78.2
Ours w/o Faith	86.0	21.2	3.4	16.8	49.1	61.9	16.4	79.1
Ours w/o CS-MIB	85.9	20.8	3.3	16.6	48.8	61.7	16.5	79.8
Ours	**86.5**	**23.5**	**3.8**	**17.4**	**50.6**	**62.8**	**15.9**	**82.0**

**Table 2:** Automatic evaluation on e-SNLI. Higher is better for all metrics except AvgLen, where lower values indicate more concise explanations. Dist and Nov denote Distinct and Novelty, respectively. The best performance is in bold.

Method	BERTScore	CIDEr	BLEU-4	Dist-1	Dist-2	Nov-2	AvgLen	Verifier Acc.	NLI Consistency
Supervised Generator	88.7	88.0	20.0	5.4	22.6	35.3	12.3	87.2	84.5
BottleSum	86.0	38.0	6.0	5.4	24.0	32.5	18.7	72.1	70.8
Prompting	85.8	17.3	4.5	3.6	15.7	36.3	27.6	69.5	67.9
Prompting–Filter	86.4	19.5	5.4	3.4	16.9	34.6	13.0	73.8	71.2
EIB	87.1	42.5	5.9	5.7	22.5	37.0	15.3	78.6	76.9
Ours w/o Faith	87.3	43.8	6.1	5.9	23.2	37.5	15.0	79.5	77.8
Ours w/o CS-MIB	87.2	43.1	6.0	5.8	22.9	37.3	15.1	80.3	78.4
Ours	**87.8**	**46.5**	**6.6**	**6.2**	**24.5**	**38.4**	**14.6**	**83.6**	**81.5**

**Table 3:** Evaluation with naturally generated LLM explanations on ECQA. Higher is better for all metrics except AvgLen (average length), where lower values indicate more concise explanations. The best performance is in bold.

Method	BERTScore	CIDEr	BLEU-4	AvgLen	Verifier Acc.
Prompting	84.7	18.2	3.6	27.8	72.4
EIB	86.1	21.5	3.8	16.9	78.6
Ours	**86.8**	**23.1**	**4.0**	**15.8**	**81.3**

**Table 4:** Evaluation with naturally generated LLM explanations on e-SNLI. Higher is better for all metrics except AvgLen (average length), where lower values indicate more concise explanations. The best performance is in bold.

Method	BERTScore	CIDEr	BLEU-4	AvgLen	Verifier Acc.	NLI Consistency
Prompting	86.3	39.8	5.8	15.7	70.4	78.2
EIB	87.4	44.1	6.3	15.2	79.1	80.3
Ours	**88.0**	**46.8**	**6.6**	**14.7**	**83.0**	**82.1**

**Table 5:** Ablation study on ECQA.

Method	BERTScore	Distinct-2	AvgLen	Verifier Acc.
Full Model	86.5	50.6	15.9	82.0
w/o Faithfulness	86.0	49.1	16.4	79.1
w/o CS-MIB	85.9	48.8	16.5	79.8
w/o Preservation	84.9	33.5	19.8	73.2
w/o Bottleneck	84.6	22.0	23.0	71.6

**Table 6:** Effect of the number of Gaussian mixture components on ECQA.

Components	BERTScore	CIDEr	AvgLen	Verifier Acc.
M=1	85.9	20.8	16.5	79.8
M=4	86.2	22.1	16.2	81.1
M=8	86.5	23.5	15.9	82.0
M=16	86.4	23.4	15.8	82.1

**Table 7:** Batch-size sensitivity analysis of the proposed CS-MIB on ECQA. The default batch size used in the main experiments is 32. Relative time denotes the training time per epoch normalized to the default setting.

Batch Size	BERTScore	CIDEr	AvgLen	Verifier Acc.	Relative Time
16	86.2	23.1	16.1	81.6	0.86×
32	86.5	23.5	15.9	82.0	1.00×
64	86.6	23.6	15.8	82.1	1.32×
128	86.5	23.4	15.8	81.9	1.86×

**Table 8:** Compression–faithfulness trade-off on ECQA.

β	BERTScore	CIDEr	AvgLen	Verifier Acc.
$10^{-5}$	86.0	21.2	18.7	80.5
$10^{-4}$	86.5	23.5	15.9	82.0
$10^{-3}$	85.7	20.1	12.8	78.4

**Table 9:** Human evaluation results. Scores are on a 1–3 scale, where higher is better. New Info. denotes new information.

Method	Gram.	Fact.	New Info.	Suff.	Conc.	Faith.
Human Ref.	2.98	2.96	2.92	2.84	2.74	2.86
Prompting	2.88	2.55	2.70	1.95	1.55	1.82
Prompting–Filter	2.90	2.68	2.64	2.10	1.60	1.96
EIB	2.95	2.76	2.74	2.25	2.40	2.18
Ours	2.96	2.82	2.78	2.43	2.52	2.46

## Data Availability

The datasets analyzed in this study are publicly available from their respective sources. ECQA is available at https://github.com/IBM/ecqa (accessed on 4 July 2026), and e-SNLI is available at https://github.com/OanaMariaCamburu/e-SNLI (accessed on 4 July 2026).
